# Bacteremia and Antimicrobial Drug Resistance over Time, Ghana

**DOI:** 10.3201/eid1710.110327

**Published:** 2011-10

**Authors:** Uwe Groß, Sylvarius K. Amuzu, Ring de Ciman, Iparkhan Kassimova, Lisa Groß, Wolfgang Rabsch, Ulrike Rosenberg, Marco Schulze, August Stich, Ortrud Zimmermann

**Affiliations:** University Medical Center, Göttingen, Germany (U. Groß, L. Groß, O. Zimmermann);; Holy Family Hospital, Nkawkaw, Ghana (S.K. Amuzu);; St. Francis Xavier Hospital, Assin Foso, Ghana (R. de Ciman);; St. Martin de Porres Hospital, Eikwe, Ghana (I. Kassimova);; Robert Koch Institute, Wernigerode, Germany (W. Rabsch);; Helios Hospital, Northeim, Germany (U. Rosenberg);; Medical Mission Institute, Würzburg, Germany (M. Schulze, A. Stich)

**Keywords:** bacteria, fever of unknown origin, FUO, bloodstream infection, bacteremia, typhoid fever, *Salmonella enterica* serovar Typhi, antibiotic resistance, antimicrobial resistance, chloramphenicol, ciprofloxacin, Ghana, dispatch

## Abstract

Bacterial distribution and antimicrobial drug resistance were monitored in patients with bacterial bloodstream infections in rural hospitals in Ghana. In 2001–2002 and in 2009, *Salmonella enterica* serovar Typhi was the most prevalent pathogen. Although most *S. enterica* serovar Typhi isolates were chloramphenicol resistant, all isolates tested were susceptible to ciprofloxacin.

In Africa, fever is usually a synonym for malaria. However, evidence exists that a large proportion of fever of unknown origin (FUO) can be attributed to bacterial bloodstream infections (BBSI). Although *Staphylococcus aureus* is the predominant cause of BBSI in industrialized countries (*1*), in African countries such as Ghana or Kenya, gram-negative bacteria are identified most often in BBSI (*2**,**3*). Furthermore, because of a lack of epidemiologic data, FUO in Africa is often treated sequentially, first with antimalarial drugs and then, until some years ago, with antimicrobial drugs such as chloramphenicol. This strategy has often been ineffective (*4*).

## The Study

In 2000 in hospitals in Ghana, we began to establish bacteriologic laboratories, which since then have participated in a biannual quality control program. For this quality control, 3 encoded bacterial species and their resistance to various antimicrobial drugs must be correctly identified. Three of these hospitals took part in comparative epidemiologic studies of FUO during October 2001–April 2002 and again during August–September 2009 with the objective of establishing a rational treatment approach (Figure). The hospitals were located in Eikwe, a coastal village that has a rural population of ≈2,000 residents; Assin Foso, which is on a regional traffic route and has a rural/urban population of ≈15,000 residents; and Nkawkaw, which is on the national traffic route that connects Accra with Kumasi and has an urban population of >45,000 residents.

**Figure:**
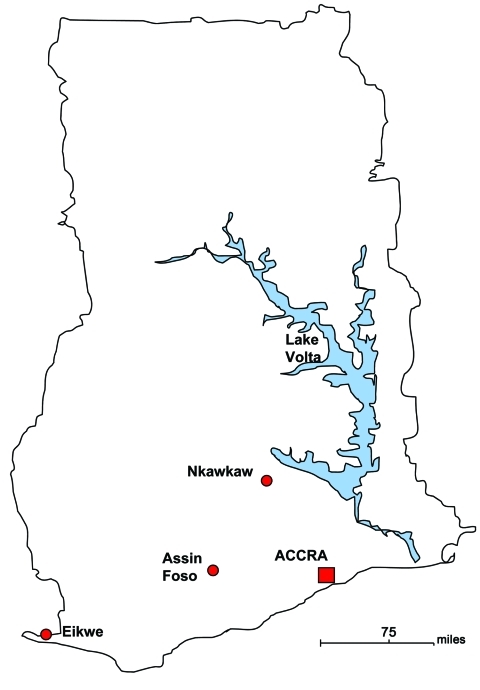
Location of populations in a study of bacteremia and antimicrobial drug resistance over time, Ghana.

This study was approved by the ethical committee of the University Medical Center, Göttingen, Germany, and the participating hospitals in Ghana. The study design, patient selection, and diagnostic approaches were identical in both study periods; FUO was defined as fever >38.5°C of >1 week’s duration without a clear clinical or organ-specific diagnosis. During the first study period, 409 patients with a wide range of ages (interquartile range 26 years) were investigated. The second study period included 258 patients with a similar age distribution (interquartile range 27 years).

Blood film microscopy was used for malaria diagnosis. Bacteremia was determined by blood cultures; 2 mL or 5 mL of blood was incubated in 20 mL or 50 mL of locally made brain–heart infusion broth for <7 days at 37°C. Gram stains and subcultures on chocolate agar were performed after 24 h, 72 h, or when the media became turbid. Bacterial differentiation, according to good laboratory practice, and susceptibility testing by disk diffusion following National Committee for Clinical Laboratory Standards criteria (*5*) was done in Africa, and susceptibility testing that included quinolone susceptibility was confirmed by broth microdilution at the University Medical Center, Göttingen. Respective tests for species differentiation were also repeated in Göttingen. The Vi phage typing scheme from the Colindale Institute London was used for *Salmonella* spp. typing (*6*).

Of the 212 bacterial isolates recovered from the blood cultures in the first study period, 145 (68.4%) indicated a putative agent of septicemia (Table 1). *Salmonella enterica* was identified in 100 (69.0%) of all pathogen-positive blood cultures, with *S. enterica* serovar Typhi accounting for 59 (40.7%). Although the 2001 National Guidelines of Ghana listed chloramphenicol as first choice for treating typhoid fever, >80% of all bacteria identified (88.3% of all *S*. *enteric* serovar Typhi) were resistant to this drug. However, ciprofloxacin proved effective against most bacteria, especially against *S. enterica* serovar Typhi (Table 2). Thus, in 2004, the national guidelines replaced chloramphenicol with ciprofloxacin for treating typhoid fever (*7*).

**Table 1:** Comparative monitoring of bloodstream infections, Ghana, 2001–2002 and 2009*

Variable	July 2001–April 2002			July–September 2009	
	Total	Positive for *Plasmodium* spp.		Total	Positive for *Plasmodium* spp.
No. patients with fever of unknown origin	409	NA		258	NA
No. *Plasmodium* spp. positive/total no. tested (%)†	85/354 (24.0)	NA		75/177 (42.4)	NA
Total no. bacterial isolates	212	51 (24.1)		99	14 (14.1)
Skin flora contaminants	67	24 (35.8)		51	10 (19.6)
Potential pathogens	145 (100.0)	27 (18.6)		48 (100.0)	4 (8.3)
*Salmonella enterica* serovars	100 (69.0)	20 (20.0)		24 (50.0)	4 (16.7)
Typhi	59 (40.7)	11 (18.6)		15 (31.3)	2 (13.3)
Paratyphi	1 (0.7)	NF		0	NF
Nontyphoid‡	40 (27.6)	9 (22.5)		9 (18.8)	2 (22.2)
*Staphylococcus aureus*	16 (11.0)	3 (18.8)		3 (6.3)	NF
*Enterobacteriaceae* other than *Salmonella* spp.	10 (6.9)	1 (10.0)		8 (16.7)	NF
*Pseudomonas* spp.	7 (4.8)	2 (28.6)		5 (10.4)	NF
Other§	12 (8.3)	1 (8.3)		8 (16.7)	NF

*Values are no. (%) except as indicated. The total number of potential pathogens for each study period was set as 100%. Coagulase-negative staphylococci, microcooci, and bacilli were judged as skin flora contaminants. The ratio of *Plasmodium* spp.–positive patients in regard to bacterial species respective groups is indicated. NA, not applicable; NF, *Plasmodium* spp. not found. †Blood film for *Plasmodium* spp. was done in most but not all cases. If the clinical situation, patient history, or blood count strongly indicated bacterial infection, blood culture was taken as first diagnostic approach. ‡*S. enterica* serovar Enteritidis and serovar Typhimurium. §Including *Streptococcus* spp., *Enterococcus* spp., *Acinetobacter* spp.

**Table 2:** Ratio in percentages of antimicrobial drug–resistant bacterial isolates obtained from patients with bacterial bloodstream infections, Ghana, 2001–2002 and 2009*

Bacteria type and years	PEN	OXA	AMP	CEF	GEN	SMX	CMP	CIP
*Salmonella enterica* serovar Typhi								
2001–2002			93.3	1.7	0	86.7	88.3	0
2009			100	0	0	100	100	0
Nontyphoid *Salmonella* spp.								
2001–2002			100	20.0	12.5	90.0	82.5	0
2009			100	0	0	88.9	77.8	0
*Enterobacteriaceae* other than *Salmonella* spp.								
2001–2002			100	50.0	60.0	80.0	80.0	0
2009			100	87.5	37.5	62.5	50.0	50.0
Nonfermenters								
2001–2002			91.7	75.0	16.7	41.7	100	0
2009			100	100	15.4	53.8	92.3	0
*Staphylococcus aureus*								
2001–2002	81.3	0	81.3		0	0	68.8	
2009	100	0	100		0	0		
All bacteria								
2001–2002			93.6	18.9	10.7	72.1	84.3	0
2009			100	41.7	10.4	72.9	84.4	8.9

*Blank cells indicate no testing performed. PEN, penicillin; OXA, oxacillin; AMP, ampicillin; CEF, cefuroxime; GEN, gentamicin; SMX, trimethoprim/sulfamethoxazole; CMP, chloramphenicol; CIP, ciprofloxacin.

To analyze the influence of ciprofloxacin on pathogen distribution and antimicrobial drug resistance in BBSI, in 2009 we initiated a follow-up study. During the second study period, pathogenic bacteria were identified in 48 (48.5%) of 99 blood cultures; the rate of *Plasmodium*-positive patients was significantly higher (42.4% vs. 24.0%, p<0.0001; Table 1). *S. enterica* was found in 50% (24/48) of all pathogen-positive blood cultures with *S. enterica* serovar Typhi remaining the most prevalent species (Table 1). Sampling was done during different months in the 2 study periods; however, these covered mainly the dry seasons. Although seasonal differences might have had an effect on the pathogen distribution, our finding is in accordance with results from other tropical countries (*8**,**9*). In addition, although our study regions were 75–150 miles away from each other, and the hospitals were localized in villages or cities which differ notably with regards to population and structure, most *S. enterica* serovar Typhi isolates belonged to phage type D1. Therefore, the spread of a clonal bacterial population within Ghana cannot be discounted.

Although an extraordinarily high percentage of chloramphenicol resistance was obvious, this drug still was considered the first choice treatment for typhoid fever in 2001 in Ghana. Therefore, the high rate of *S. enterica* serovar Typhi was not unexpected. Similarly, 91.7% of all *S. enterica* were resistant to chloramphenicol in 2009 (Table 2). In both study periods, second-line antimicrobial agents, e.g., trimethoprim/sulfamethoxazole or ampicillin, also showed a high rate of resistance. This finding was in agreement with those of other studies from nonindustrialized countries (*10*). The rate of cefuroxime-resistant bacteria increased from 18.9% to 41.7% because of a higher percentage of cefuroxime-resistant enterobacteriaceae other than *Salmonella* (50.0% vs. 87.5%, Table 2).

In 2001–2002, most bacteria were susceptible to ciprofloxacin (Table 2), as had been shown for *S. enterica* from blood cultures of Nigerian patients (*11*). In contrast, for *S.* *enterica* serovar Typhi isolated in 1997–1999 in Kenya, MICs of ciprofloxacin were noticeably higher than for those strains isolated during 1988–1993 (*12**,**13*).

Although ciprofloxacin proved to be effective against *S. enterica* in our study, the resistance rate of enterobacteriaceae other than *S. enterica* against this quinolone increased from zero in 2001–2001 to 50.0% in 2009 (Table 2). Methicillin-resistant *S. aureus* was not identified as a cause of BBSI during either period.

When we assessed the situation in individual regions, notable differences were obvious. Comprising 47.5% of all BBSI, typhoid fever was most prevalent in Assin Foso in 2001–2002. In contrast, not even 1 case occurred in 2009. Analyzing the situation in that urban area, the following conditions were found: 1) sanitation was improved; 2) additional toilets were established; 3) ciprofloxacin was widely used in hospital for treating infections; and 4) ciprofloxacin was easily available at local street traders. Although the broad application of ciprofloxacin has to be critically discussed, the observed absence of typhoid fever in Assin Foso is impressive.

In contrast, *S.* *enterica* serovar Typhi was isolated from 27.8% of cases of BBSI in Eikwe in 2001–2002 and remained at a high rate of 30.2% in 2009. In this small fishing village, the situation differed noticeably from that in Assin Foso. Although additional toilets had been constructed, sanitation was not much improved; most residents still used the beach for defecation. In addition, ciprofloxacin was not extensively prescribed in the hospital and was not available at local street traders. Thus, educational programs to encourage use of public toilets plus adequate prescription of ciprofloxacin might help control typhoid here in the future.

## Conclusions

Although Ghana implemented several measures to control typhoid, our study found that, depending on the region, *S. enterica* serovar Typhi remains the most prevalent bacterial species causing BBSI. This finding is in agreement with a recent study from the Ashanti region, where 12.4% of BBSI were caused by *S. enterica* serovar Typhi (*14*). In addition, emergent ciprofloxacin resistance has been described in Accra, the capital of Ghana (*15*). Therefore, the implementation of bacteriologic diagnosis should be considered even in smaller hospitals in a rural African setting to monitor pathogen distribution and resistance rates.
